# Porcine Neonatal Pancreatic Cell Clusters Maintain Their Multipotency in Culture and After Transplantation

**DOI:** 10.1038/s41598-018-26404-6

**Published:** 2018-05-29

**Authors:** Wan-Chun Li, Chen-Yi Chen, Chen-Wei Kao, Pei-Chun Huang, Yi-Ta Hsieh, Tz-Yu Kuo, Tsai-Ying Chen, Hao-Yuan Chia, Jyuhn-Huarng Juang

**Affiliations:** 1 Institute of Oral Biology, School of Dentistry, National Yang-Ming University, Taipei, Taiwan; 2 Department of Dentistry, School of Dentistry, National Yang-Ming University, Taipei, Taiwan; 3 Genome Research Center, National Yang-Ming University, Taipei, Taiwan; 4 Division of Endocrinology and Metabolism and Center for Tissue Engineering, Chang Gung Memorial Hospital, Taoyuan, Taiwan; 5 Department of Medicine, College of Medicine, Chang Gung University, Taoyuan, Taiwan

## Abstract

Ductal epithelium is primarily detected in porcine neonatal pancreatic cell clusters (NPCCs) bearing grafts, suggesting that transplants might exhibit progenitor-like phenotypes. Here we found that soon after NPCC isolation, PDX1^+^/insulin^−^ and SOX9^+^ pancreatic progenitor-like cells dramatically increased while dual-hormonal progenitor-like cells were routinely observed in NPCC culture. After transplantation (Tx), insulin^+^ cells increased and PDX1^+^ and SOX9^+^ cells gradually decreased in both non-diabetic (NDM) and streptozotocin-induced diabetic (DM) grafts over 2 months. Strikingly, a significantly higher percentage of insulin^+^ cells were detected in 9-day and 16-day, but not in 23-day, 30-day and 60-day grafts implying that hyperglycemia could only facilitate NPCC-derived β cells early post-Tx. A higher percentage of NPCC-derived β cells in early DM grafts was determined via an enhanced neogenic differentiation based on the detection of insulin^+^ cells budding out from PDX1^+^/SOX9^+^ epithelium. Interestingly, a drop in SOX9^+^ progenitor-like cells was detected 16 days post-Tx in DM grafts whilst PDX1^+^ cells do not show a significant difference until 60 days post-Tx between DM and NDM grafts, demonstrating that distinct progenitor-like populations fuel new β cells post-Tx. In conclusion, PDX1^+^/SOX9^+^ cells could be quickly activated after NPCC isolation, maintain their multipotency in culture and differentiate into new β cell post-Tx.

## Introduction

Patients with Diabetes Mellitus (DM) often exhibit reduced pancreatic β-cell mass and insulin deficiency. While Type 1 diabetic patients (T1D) often proceed to be insulin-dependent, poorly controlled glycaemia due to unmatched onset and duration of injected insulin is frequently detected^1^. Insulin replacement by pancreas and islet transplantation (Tx) has been considered the most promising clinical procedure for precise glycemic control. Although the progression of human islet Tx has achieved insulin independence in T1D, most successful cases require continuous administration of immunosuppressant drugs and multiple transplantations to maintain normoglycaemia, revealing a major obstacle for the procedure^2^. To overcome this issue, numbers of surrogate β-cells, including embryonic/adult pluripotent stem cells (PSC), derived β-like cells and xenogenic islets from other animal species, are considered^3^.

Neonatal porcine pancreatic cell clusters (NPCCs) have been long utilized as an ideal xenogenic source for Tx to ameliorate hyperglycaemia due to their relatively easy isolation and culture procedure as well as great *in vitro* growth potential^4^. Previous studies show that NPCCs were capable of restoring normoglycaemia in diabetic animals, which are mainly due to β cell expansion and differentiation of residing islet precursors into β cells^5,6^. Nevertheless, the fact that NPCCs could reverse hyperglycemia in diabetic mice only until 2 months post-Tx implies that NPCCs are rather immature and possess poor glucose-responsive insulin secretion even though NPCCs could secrete significant quantities of insulin in response to a steady-state glucose challenge *in vitro*^4,7–9^.

Series of studies have tested the role of potential islet differentiation triggers such as glucagon-like peptide 1 (GLP-1)^10^, cholecystokinin (CCK)^11^ and insulin-like growth factor-1 (IGF-1)^12^ in promoting maturation of fetal islet-bearing grafts either *in vitro*^13,14^ or *in vivo*^11,15^ aiming to shorten the latent period between Tx and reversal of hyperglycemia. The results were inconsistent and showed a minimal improvement of Tx outcomes in response to these maturation promoters, further arguing a requirement to clarify cellular identity of initial transplants as well as to define dynamic molecular basis regarding how fetal islets differentiate in culture and after Tx.

In our current study, we therefore examined the cellular changes of endocrine lineages of cultured NPCCs and NPCC grafts in a time-course manner using either RT-PCR or quantitative immunofluorescence staining analysis. Based on recent advances in understanding the molecular hierarchy in pancreatic organogenesis^16^ and previous studies showing that 57% of *in vitro* cultivated NPCCs exhibited primarily epithelial progenitor-like phenotypes^4^, we determined the expression of progenitor markers Pancreatic and duodenal homeobox 1 (PDX1) and Sex-determining region Y-box containing gene 9 (SOX9) in cultured NPCCs and NPCC grafts from both nondiabetic (NDM) and streptozotocin-induced diabetic (DM) receipt mice to better delineate a potential progenitor mediated β cell differentiation as well as a hyperglycemia mediated effect for porcine islet precursor-like cells.

## Results

### Enrichment of Endocrine Cells in Cultured NPCCs

**Figure 1:**
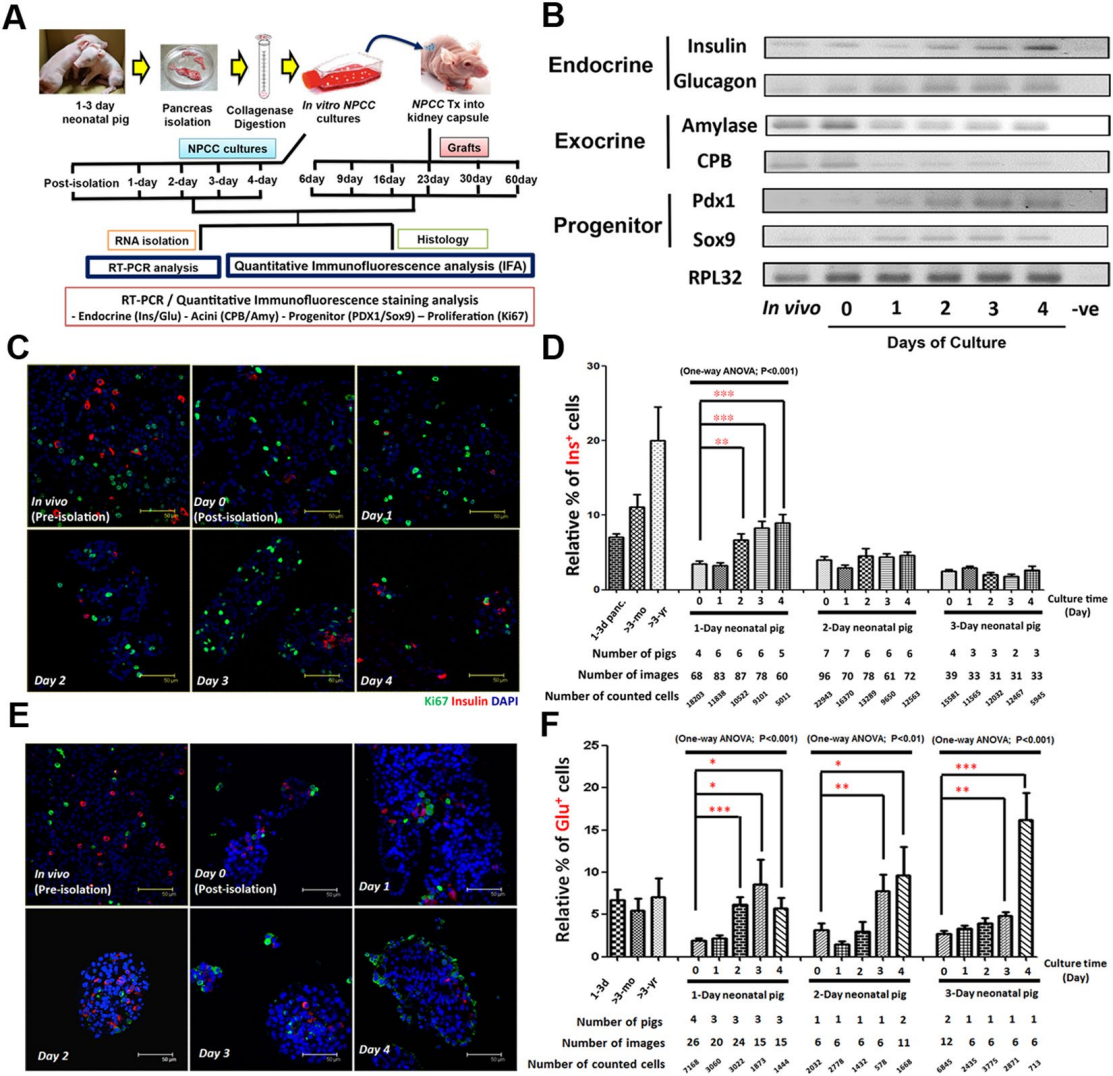
Induction of endocrine and progenitor program in NPCC cultures. (**A**) Experimental scheme of current study. (**B**) Semi-quantitative RT-PCR analysis indicated an elevated mRNA expression for endocrine markers insulin and glucagon and progenitor markers Pdx1 and Sox9 in NPCC cultures. Decreased mRNA expression of exocrine enzymes CPB and amylase, in contrast, was down-regulated during NPCC cultures. Quantitative immunofluorescence staining analysis (qIFA) for Ki67/glucagon (green) and insulin (Red) showed (**C**,**D**) enriched insulin^+^ cells and (**E**,**F**) upregulated glucagon^+^ cells in NPCC culture over 4 days. 1–3d panc: 1-day, 2-day and 3-day postnatal pig pancreata (N = 3 for each time point); >3 month: 3-month-old pig pancreas (N = 1); >3 yr: 3-year-old pig pancreas (N = 2); DAPI is used to localize cell nuclei and Y-axis represented the percentages of (**D**) insulin^+^/DAPI^+^ and (**F**) glucagon^+^/DAPI^+^ cells. *p < 0.05, **p < 0.01, ***p < 0.001.

The experimental scheme was designated (Fig. 1A) to examine changes of mRNA and protein expression in endocrine, exocrine and progenitor-like cells in cultured NPCCs and NPCC grafts in NDM or DM mice. Under our culture condition, we found increased dead cell debris in 8-day cultured NPCCs (Supplemental Fig. 1A). Consistent with a recent finding^17^, the detection of higher level of reactive oxygen species (ROS) might serve as a potent trigger for upregulated cytotoxicity in 1- to 4-day NPCC cultures (Supplemental Fig. 1B). To avoid potential adverse influence from apoptotic cells, we therefore decided to focus on investigating molecular cues in 1- to 4-day NPCC culture while utilizing 3-day cultured NPCCs for *in vivo* transplantation experiments.

In agreement with previous reports^13^, we observed an upregulation of insulin mRNA in cultured NPCCs; semi-quantitative RT-PCR analysis demonstrated that expression of both insulin and glucagon mRNA was increased in 1- to 4-day cultured NPCCs in a time-dependent manner. On the contrary to 1- to 3-day postnatal pancreata tissues, the expression of exocrine genes including carboxypeptidase B (CPB) and amylase was downregulated over time in cultured NPCCs (Fig. 1B). Quantitative immunofluorescence analysis (qIFA) was further performed to determine the changes of insulin^+^ cells, glucagon^+^ cells, somatostatin (SS)^+^ cells and pancreatic polypeptide (PP)^+^ cells in cultured NPCCs. It was found that percentages of insulin^+^, glucagon^+^ (Fig. 1C–F) and PP^+^ cells, but not SS^+^ cells (Supplemental Fig. 2), were increased over a 4-day culture compared with freshly isolated NPCCs, implying that our NPCC culture condition could enrich endocrine cell lineage and suppress exocrine cells. Moreover, NPCCs from 1-day neonatal pigs exhibited a more dominant differentiation capacity into insulin^+^ cells during culture (Fig. 1D), although not much difference was detected for glucagon^+^ cells (Fig. 1F). To better define which cell population is missing during NPCC isolation, we quantitatively determined changes of amylase^+^ exocrine cells in 1- to 4-day NPCC cultures. The results showed a quick loss of amylase^+^ cells in pre-culture NPCC isolate (Day 0) and the percentages of amylase^+^ cells remained low over 4 days of NPCC cultivation indicating that the dead cells during NPCC culture were mainly exocrine cells (Supplemental Fig. 3). It is also noteworthy that, regardless of ages of neonatal pigs, endocrine cell fate seems to dedifferentiate in response to NPCC isolation procedure as insulin^+^, glucagon^+^ and PP^+^ cells are decreased after isolation compared to neonatal pig pancreas (Fig. 1D,F and Supplemental Fig. 2C) reflecting that NPCCs possess great plasticity for cellular dedifferentiation/redifferentiation. Taken together, our results indicated that isolation might redirect NPCCs to a more naïve state and then recapitulate endocrine differentiation following a normal developmental program *in vitro*.

### NPCC Isolation Quickly Activated Multi-hormonal/PDX1^+^/SOX9^+^ Progenitor-like Cells

**Figure 2:**
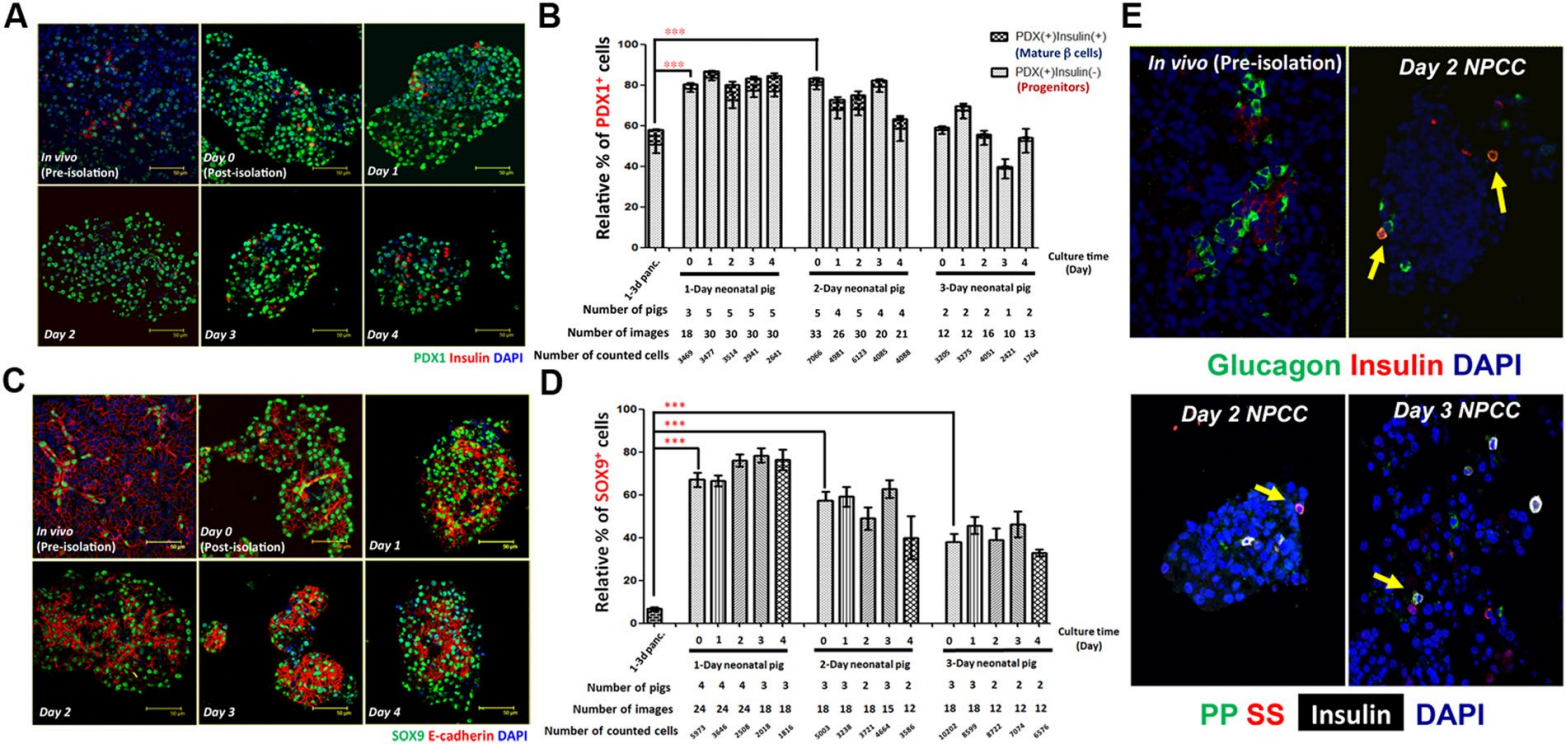
NPCC isolation simply activates PDX1^+^/SOX9^+^/multi-hormonal pancreatic progenitor-like cells in cultured NPCCs. IFA for (**A**) PDX1 (green)/insulin (red) and (**C**) SOX9 (green)/E-cadherin (red) in NPCC cultures. Quantitative results showed a significant increase of (**B**) PDX1^+^ and (**D**) SOX9^+^ cell populations in freshly-isolated NPCCs, compared to neonatal pig pancreas, and maintained high percentage over 4 days in culture. NPCCs from 1- to 2-day neonatal pigs showed significantly more progenitors than those from 3-day new-born pigs; (**E**) Rare but reproducible multi-hormonal progenitor-like cells (arrows) in 2-day NPCC cultures isolated from 1-day neonatal pigs was detected by IFA. DAPI was used to localize cell nuclei. ***p < 0.001.

In addition to changing endocrine and exocrine cell populations during NPCC culture, the activation of pancreatic precursor-like cells in response to NPCC isolation was also emphasized. It was also interestingly found that the mRNA expression of pancreatic progenitor transcription factor Pancreatic and Duodenal homeobox 1 (PDX1)^18^ was greatly upregulated in cultured NPCC compared with 1–3 day neonatal pig pancreas (Fig. 1B). We further analyzed the alterations of PDX1^+^/Ins^−^ progenitor-like population and PDX1^+^/Ins^+^ mature cells in NPCC culture by qIFA (Fig. 2A). Surprisingly, it was found that the percentage of PDX1^+^/Ins^−^ pancreatic precursor-like cells significantly increased in freshly isolated NPCCs (80.6% at 0-day culture) in comparison with 1-day neonatal pig pancreas (51.9%) and maintained a high percentage over 4 days in culture (83.7%, 72.7%, 77.3% and 77.1% at 1-, 2-, 3- and 4-day culture, respectively) (Fig. 2B). It is also important to emphasize that when we delineated the differential enrichment of PDX1^+^/Ins^−^ cells, based on different ages of pig NPCCs isolated from 1- or 2-day neonatal pigs, they displayed a greater cell plasticity to dedifferentiate into a progenitor-like state over culture compared to the 3-day neonatal pigs, implying that pancreatic cell fate determination is highly dynamic at early stages after birth.

It was previously shown that SOX9 acts as a key player in maintaining the pancreatic multipotent progenitor pool throughout pancreogenesis^19^. While a very recent study interestingly elucidated that PDX1 and SOX9 cooperatively establish pancreatic lineage identity by excluding intestinal lineage-restricted transcription factors from foregut endoderm progenitor cells^20^, whether SOX9^+^ pancreatic precursor-like cells could be activated during NPCC isolation/culture was also examined (Fig. 2C). The results confirmed that both SOX9 mRNA expression (Fig. 1B) and enriched SOX9^+^ epithelial cells were detected after isolation (67.1% at 0-day culture compared to 6.8% in 1-day old pig pancreases) and over 4-day culture (78.0%, 79.7%, 80.0% and 81.3% at 1-, 2-, 3- and 4-day culture, respectively) (Fig. 2D), which might result from a great loss of acinar cells after NPCC isolation and in culture.

Previous studies in a variety of species have also shown that endocrine cells, in adult islets and during neogenesis, express more than one islet hormone during islet development in the fetal state^21,22^. With a single-cell gene-profiling method, it was found that a single islet cell could co-express 2, 3 or all islet hormone genes (e.g. insulin, glucagon, somatostatin and/or pancreatic polypeptide) in either developing or adult islets^23,24^. Consistent with previous findings, precursor-like cells, namely insulin^+^/glucagon^+^, insulin^+^/PP^+^ and insulin^+^/somatostatin^+^ co-stained cells, could be detected in NPCC isolates^25^, and rare but consistent multi-hormonal precursor-like cells were visualized in 2- and 3-day cultured NPCCs (Fig. 2E, arrows). Taken together, NPCC isolation could simply activate multi-hormonal/PDX1^+^/SOX9^+^ pancreatic progenitor-like cells and the NPCC culture condition could preserve their multipotency over time regardless of the animal age at isolation.

### NPCCs Undergo *de novo* Developmental Program Post Tx

**Figure 3:**
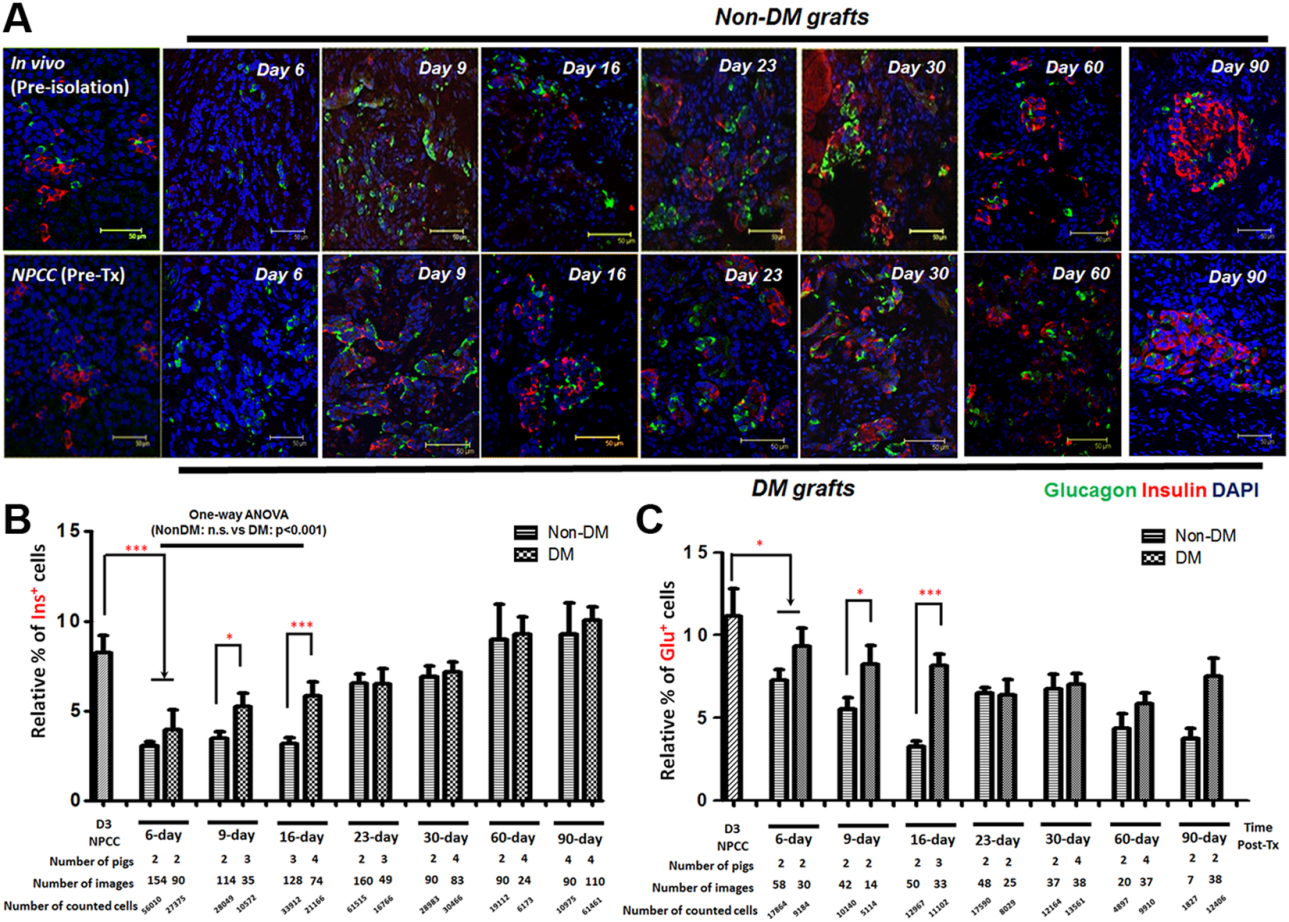
Endocrine differentiation in NPCC-bearing grafts post-Tx. (**A**) IFA for insulin (red) and glucagon (green) in NPCC-bearing grafts from NDM/DM mice. Quantitative results showed significantly increased (**B**) insulin^+^ cells and decreased (**C**) glucagon^+^ cells in grafts from DM mice when compared to NDM subject. DAPI was used to localize cell nuclei. *p < 0.05, ***p < 0.001.

While most readouts of NPCC Tx outcome were mainly focused on the gradual reversal of hyperglycemia in mice with successful grafts, our results indicated that the initial transplanted NPCCs exhibited a predominantly precursor-like cell population. As we showed in the present study that, in consistent with a previous finding^6^, a STZ-induced hyperglycemia could only be moderately ameliorated after 90 days of NPCC Tx (Supplemental Fig. 4); better understanding of cellular and molecular changes of NPCCs post-Tx would potentially be critical to define important intrinsic and external players to facilitate Tx outcome (e.g. to shorten time window of glycemic normalization post Tx)^14,26^. Furthermore, the underlying mechanisms of immune rejections upon NPCC xenotransplantation could also possibly be addressed if impacts of adjacent microenvironment of NPCCs-bearing grafts are elucidated^27^. We therefore examined the changes of insulin^+^, glucagon^+^ (Fig. 3A), Ki67^+^, PDX1^+^ and SOX9^+^ cells in grafts from NDM and DM nude mice by qIFA to determine cellular dynamics during progenitor-mediated endocrine differentiation. The data showed a gradual increase of insulin^+^ cells (Fig. 3B) and a mild drop in percentage of glucagon^+^ cells (Fig. 3C) in grafts from both NDM and DM mice over 3 months post Tx. The results indicated that NPCCs might undergo differentiation into endocrine lineage following a *de novo* developmental program post-Tx. Interestingly, a drop in insulin^+^ cells in 6-day grafts (3.01% in NDM and 3.95% in DM mice) compared to 3-day cultured NPCCs prior to Tx (8.24%), further supporting that NPCCs could reset their developmental program in response to differential environmental cues.

### Hyperglycemia Facilitated Progenitor Mediated Endocrine Maturation via Neogenesis

**Figure 4:**
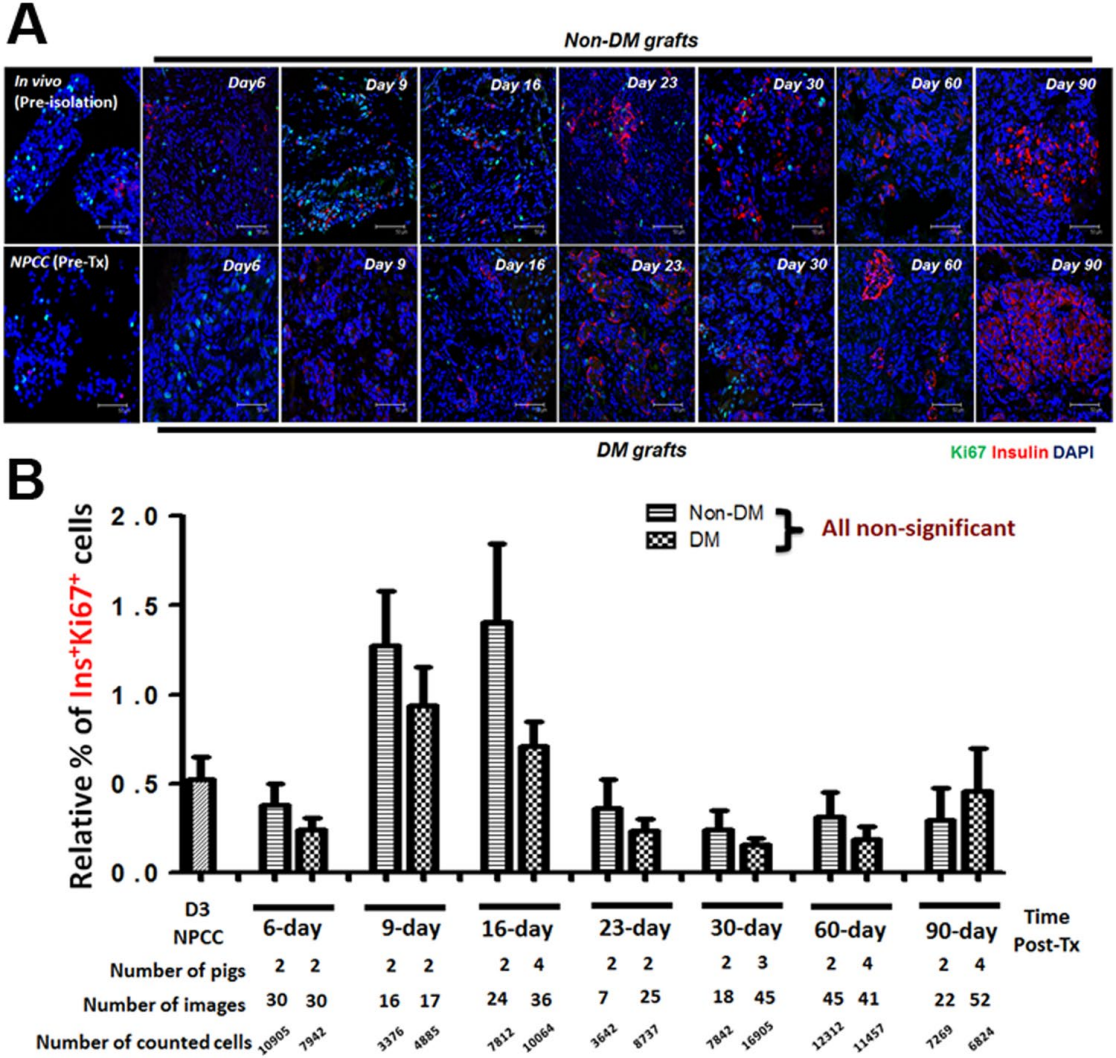
Self-replication has no significant impact to NPCC differentiation post-Tx. (**A**) IFA for Ki67^**+**^ (green) and insulin^**+**^ (red) cells in NPCC-bearing grafts from NDM and DM mice; (**B**) quantitative analysis showed no significant difference for Ki67^**+**^ β cells between NDM and DM mice. DAPI was used to localize cell nuclei. n.s.: non-significant.

**Figure 5:**
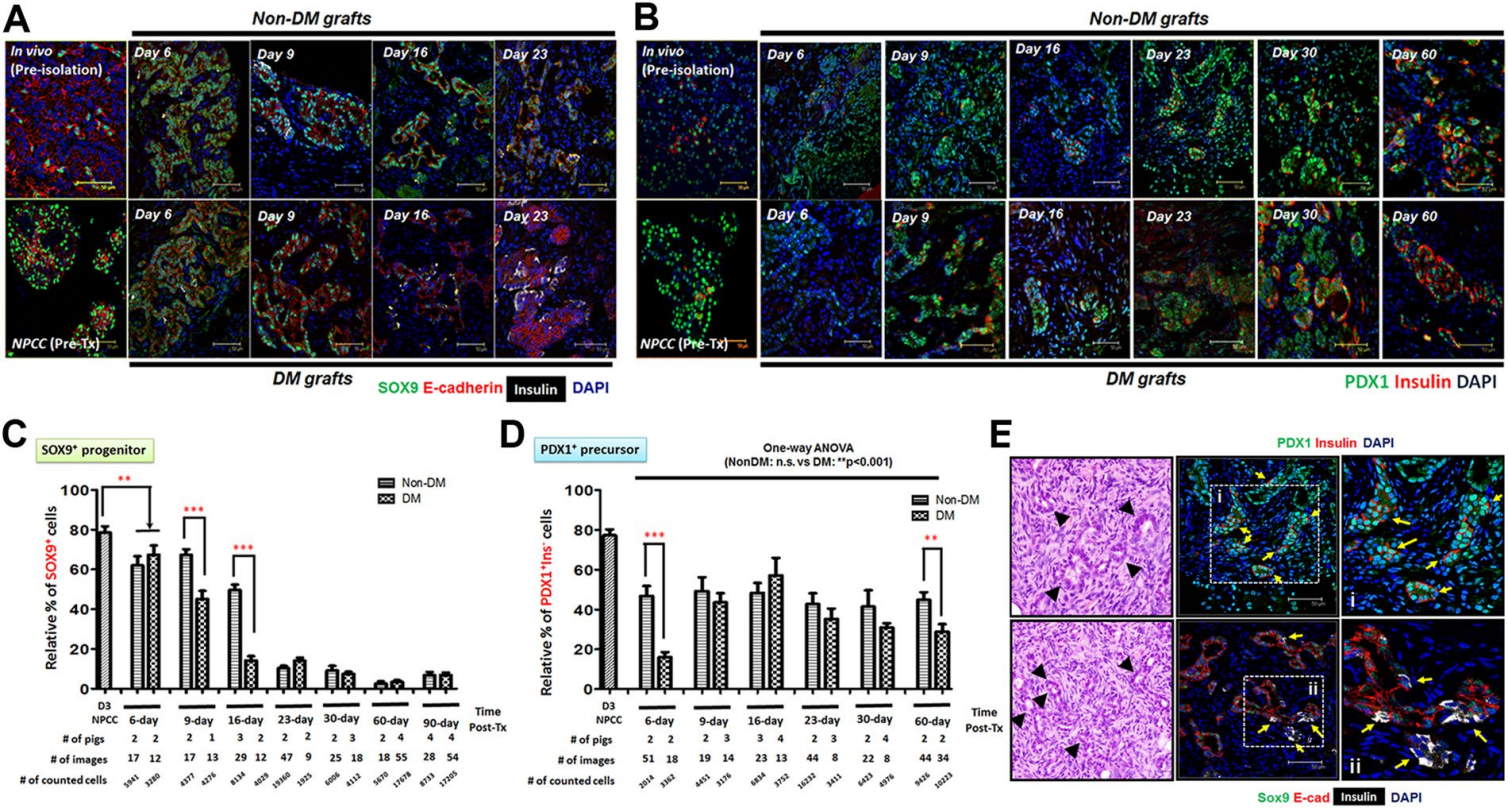
SOX9^+^ and PDX1^+^ progenitor-like cells differentiate into new β cells via neogenesis post-Tx. IFA for (**A**) PDX1 (green)/insulin (red) and (**B**) SOX9 (green)/E-cadherin (red) in NPCC-bearing grafts from NDM and DM mice. (**C**) Quantitative results showed a significant decrease of PDX1^+^ and SOX9^+^ cells post-Tx. Sox9^+^ precursors decreased faster in DM than in NDM grafts after 16 days post-Tx while PDX1^+^ cells only showed a significant difference between NDM and DM grafts at 6- and 60-day grafts. (**E**) Neogenesis underlies NPCC progenitor-mediated β cell differentiation post-Tx. H&E staining showed small progenitor-like ducts (arrowheads) in NPCC-bearing grafts from DM mice 16 days post-Tx. IFA indicated Insulin^+^ cells (red or white, arrows) budding out of PDX1^+^/Sox9^+^ (green) epithelium. i and ii: enlarged images of the neogenic β cells in dotted boxes. DAPI was used to localize cell nuclei. **p < 0.01, ***p < 0.001.

A previous study showed that increased β cell mass in NPCC Tx was contributed by both an enhanced proliferation of Ki67^+^ β cells and an upregulated maturation of CK7^+^insulin^+^ protodifferentiated cells^25^. Moreover, it was found that cured diabetic recipients had significantly higher β cell proliferation compared with normoglycaemic recipients^6^. The lack of characterized progenitor markers at that time, however, limited further identification of potential hyperglycemic effects on progenitor-mediated islet maturation in NPCCs-bearing grafts. In the present study, the temporal changes for PDX1^+^/SOX9^+^ progenitor-like cells and its corresponding β cell differentiation was therefore examined to quantitatively trace PDX1^+^/SOX9^+^ precursor-like cells in grafts of NDM and DM mice. The results demonstrated that the relative β cell population is greater in DM than in NDM grafts within the first 2 weeks post-Tx but became indistinguishable over 3 months after Tx, suggesting that the normal endocrine maturation program was accelerated under DM conditions at early stages post-Tx (Fig. 3B). An increased replicating Ki67^+^ β cell population could be detected in grafts from both NDM and DM mice at an early stage post-Tx (Fig. 4A); nevertheless, the percentage of replicating β cells showed no significant difference between NDM and DM grafts over 3 months post-Tx suggesting that self-replication might not dominantly play a role in regulating insulin^+^ cell population in DM grafts (Fig. 4B). Neogenesis, another potential mechanism for increased β cell populations in NPCCs-bearing grafts, was therefore further analyzed. The relative number of NPCC-derived SOX9^+^ (Fig. 5A,C) and PDX1^+^Ins^−^ (Fig. 5B,D) progenitor-like cells rapidly decreased, by varying degrees, to levels similar to 1-day neonatal pig pancreas upon Tx accompanied by increasing β cell populations (Fig. 5A–D). Furthermore, faster decrease of SOX9^+^ precursor-like cells was noted in 9-day and 16-day DM grafts compared with NDM grafts (Fig. 5C), implying that hyperglycemia could possibly trigger NPCC progenitor-like cells to differentiate more rapidly. Indeed, only in DM grafts from 9-day (3 out of 8 mice) and 16-day (3 out of 7 mice), insulin^+^ cells budding out of PDX1^+^/SOX9^+^ epithelial progenitor-like cells could be detected (Fig. 5E). Another interesting finding in the present study is that a significantly greater percentage of PDX1^+^ progenitor-like cells could be detected at 60 days after Tx (Fig. 5D), revealing that there is (1) a more significant role of PDX1 in controlling β cell maturity and (2) NPCC-derived SOX9^+^ and PDX1^+^ progenitor-like cells differentially fuel new β cells post-Tx in DM animals.

## Discussion

In the current study, we demonstrated that when accompanied by enriched endocrine cells, the PDX1^+^/SOX9^+^ pancreatic progenitor-like cells could be activated during NPCC isolations and NPCC-derived precursor-like cells could maintain their multipotency in cultivation. This observation is consistent with previous reports which showed that after 3- and 9-day cultures, the percentage of α and β cells significantly increased compared to the initial isolation and the duct-like structures were primarily detected in NPCC cultures^4^. Additionally, in agreement with previous studies^6^, upon Tx, the progenitors undergo self-replication (detection of Ki67^+^ cells) and neogenesis (detection of Ins^+^ cells budding out of SOX9^+^ epithelium) in both NDM and DM receipt mice. Interestingly, further analysis delineated that a diabetic environment could potentially trigger progenitor mediated β cell differentiation while PDX1^+^ and SOX9^+^ precursor-like cells act differentially to regenerate newly formed β cells in DM grafts compared with the NDM group. Our results, for the first time, provided the mechanistic evidence showing that PDX1^+^/insulin^−^ and SOX9^+^ porcine pancreatic progenitor-like cells could be markedly propagated and undergo maturation after NPCC isolation and post-Tx, respectively.

The reasons why and how routine isolation could quickly stimulate NPCC-derived PDX1^+^/SOX9^+^ pancreatic progenitor-like cells *in vitro* remain in question. Although factors involved in an activation of pancreatic precursor-like cells are rarely discussed, the liberation of pancreatic tissue integrity by enzymatic digestion and mechanical shaking could be contributing to this outcome. Indeed, the progenitor-associated regeneration was reported in different animal models (e.g. partial pancreatectomy or pancreatic ductal ligation) mainly under conditions of ablation for pancreatic tissue architectures^28,29^ implying that tissue disruption could be a key trigger to “wake” pancreatic precursors for tissue homeostatic maintenance. From a physiological standpoint, a recent study showed that non-resolving inflammation, resulting from elevated interferon-γ produced by neutrophils and increased induced nitric oxide synthase (iNOS) secreted from pro-inflammatory macrophages, led to impaired caerulein-induced pancreatic regeneration, further providing a potential impact of inflammation for pancreatic progenitor activation in response to pancreatic injury^30^.

The molecular mechanisms underlying pancreatic endocrine development have been extensively studied over decades^16^. In general, genetically deficient mice were utilized to define regulations of intracellular molecules (i.e. transcription factors) and external soluble cues from surrounding environments in controlling pancreatic endocrine development. In early development, the PDX1 protein marks the pancreatic anlage starting at E8.5 in mice^31^. Global knockout of *pdx1* resulted in the absence of a pancreas at birth in mice^32^ and pancreatic agenesis in humans^33^. A hypomorphic allele of PDX1 resulted in a remnant pancreas with ductal and acinar cells, but a relative lack of endocrine cell development^34^ indicating its importance in regulating early pancreatic genesis. In the late gestational and mature pancreas, PDX1 expression is restricted primarily to insulin-producing β cells with low-level expression in subpopulations of acinar and duct cells^35^. Complete or near-complete deletion of PDX1 in late embryonic^36^ or adult^37^ mice led to a later diabetic phenotype suggesting a dramatically different requirement for PDX1 gene dosage in the developing pancreas vs. mature β cells, the latter of which may require more tightly regulated transcription or translation. In addition, accumulating evidence, acquired primarily from studying knockout and transgenic mice, has revealed that SOX9 serves as a critical molecule for pancreatic progenitors as well as for maintenance of embryonic and adult pancreatic ductal state^19,38,39^. SOX9 protein is highly expressed in both emerging pancreatic buds at E9.5 in mice when they comprise exclusively of multipotent progenitors^40^. Moreover, the SOX9 paralog SOX9b is expressed in early zebrafish hepatopancreatic endoderm, marking both the base of the dorsal pancreas and the prospective ventral pancreas^41^. In humans, a recent immunohistochemistry based analysis demonstrated that SOX9 is weakly costained with PDX1 in prospective duodenal-pancreatic endoderm by Carnegie Stage (CS) 12 (equivalent to ~E9–9.5 in mouse embryogenesis). By CS13, SOX9 is strongly expressed in the dorsal and ventral pancreatic buds and is maintained through CS16 in the branched pancreatic epithelium^42^. Nevertheless, most investigations were focused on earlier processes, such as cell commitment and migration of precursors, whereas the key regulatory molecules in driving pancreatic precursors into functional β cells are still poorly identified. Although the first human β cell line EndoC-βH1 was recently established^43^, making it possible to investigate human islet physiology without species bias, the genetic manipulation restricts its use to define *bone fide* β cell maturation triggers using this cell model. It therefore becomes essential to develop a stable *in vitro* system similar to human pancreatic progenitors or immature β cell culture. Based on our present data, short-term NPCC culture as well as long-term NPCC-bearing grafts could be ideal platforms to more convincingly test insulinotropic agents for porcine/human pancreatic precursor-like cells from postnatal pancreas. Previous studies, however, still failed to enhance mature β-cell characteristics in NPCC culture supplemented with GLP-1^15^ or IGF-1 alone^13^ compared with control group *in vitro*; or to improve Tx outcome by shortening the latent period between Tx and reversal of hyperglycemia *in vivo*^13,14^. These results indicated a great demand to profile molecular changes of PDX1^+^/SOX9^+^ and insulin^+^ cells in NPCC-bearing grafts dynamically. To this end, a genetic heterogeneity in pancreatic β cells should also be taken into consideration to more precisely correlate molecular cues of progenitor-derived β cells with their maturity.

Another important finding of the present study is the discovery of DM mediated machinery of porcine pancreatic precursor-mediated β cell differentiation. The role of elevated circulating blood glucose in controlling maturation of islet progenitor-like cells into functional β cells was rarely discussed since it is extremely difficult to delineate the cause-and-effect relationship between hyperglycaemia and β cell differentiation in transplantation setting. Interestingly, in agreement with our findings, a previous study did show that, in comparison with hyperglycaemic condition, a long-term euglycaemic exposure reduced the functional maturation of NPCC grafts^44^. It is also noteworthy that, in our experimental design, NPCC-bearing grafts only mildly rescued hyperglycaemia in DM mice providing a great condition to investigate DM based regulation for porcine pancreatic precursor-mediated β cell differentiation. However, it should be aware that NPCC-bearing grafts might undergo different differentiation process if normoglycaemia could be achieved in DM animals.

In mechanistic basis, neogenesis, rather than self-duplication, seems to play a more significant role for new β cell formation in DM NPCC-bearing grafts. To date, the origin of postnatal regenerating β cells is still in question. Most cell-lineage studies using transgenic mice have shown that new β cells, in response to various stimuli, are mainly derived from pre-existing mature β cells^45,46^; in contrast, neogenic β cells, mainly from pancreatic ductal cells, have also been routinely detected in different regeneration rodent models^29,47–49^, resulting in a long lasting debate. The human pancreas is a slow-turnover organ and accumulating evidence from recent investigations seems to reach a consensus that, in comparison with self-replication, neogenesis predominantly serves as an underlying mechanism for new-forming β cells in humans^50,51^. In agreement with findings in human subjects, NPCC-derived PDX1^+^ and SOX9^+^ progenitor-like cells both contributed, but act differentially in time, to form new β cells. SOX9^+^ precursor-like cells rapidly respond to a diabetic environment in the first 2 weeks post-Tx as the percentage of PDX1^+^ progenitor-like cells remained steady in both NDM and DM mice within the first month post-Tx; as only slightly more PDX1^+^ insulin^−^ precursor-like cells participated in generation of new β cells in DM mice at 60-day post-Tx. It is noteworthy that a significant difference for insulin^+^ cells between NDM and DM mice was only evident after the first 2 weeks post-Tx implying that SOX9^+^ epithelium in NPCCs could differentiate into insulin^+^ and glucagon^+^ cells possibly through a neogenesis-like process. If this scenario holds true, a significant increase of insulin^+^ cells in DM mice would only be detected early post-Tx since SOX9^+^ progenitor-like cells are quickly deprived in the first 2 weeks post-Tx without much supply from PDX1^+^ cells. Further studies should be focused on identifying possible molecules to facilitate differentiation efficacy of PDX1^+^ precursor-like cells into functional β cells.

Collectively, our findings showed that porcine precursor-like cells could simply be activated during NPCC isolation. NPCC-derived progenitor-like cells maintain their multipotency in culture providing a useful platform to examine maturation-promoting effects of candidate molecules, which could be used to direct human pluripotent stem cells into functional β cells. Furthermore, isolation and characterization of distinct porcine progenitor cells in NPCC culture/grafts could enhance NPCC-to-human xeno-Tx efficiency and Tx outcomes in the future.

## Methods

### Animal procedure

All animal procedures (IACUC number: 2012122001) were conducted in accordance with the Institutional Animal Care and Use Committee (IACUC), Chang Gung Memorial Hospital, Taoyuan, Taiwan. Authors confirm that all methods and protocols of animal experiments were carried out and approved by IACUC), Chang Gung Memorial Hospital, Taoyuan, Taiwan. The animal numbers, both for pigs and nude mice, used in each condition are described in each Figure accordingly. Donor pancreata were obtained from 1- to 3-day-old neonatal pigs of either gender following a previously described procedure^4,9^. Male athymic nude Balb/c mice aged 8–12 weeks, without contamination with relevant pathogens, were obtained from The National Laboratory Animal Center, Taiwan as recipients of the NPCCs. Mice were rendered diabetic by intraperitoneal injection of 190 mg/kg streptozotocin (STZ) which was freshly prepared before use. For preparation and culture of NPCCs, pig pancreata were cut into fragments of 1 to 2 mm^3^, then digested by type V collagenase (Sigma) in a water bath at 37 °C. The digest was filtered, washed and then placed in culture medium containing RPMI1640 medium supplemented with 50 mM IBMX, 0.5% BSA, 2 mM L-glutamine, 10 mM nicotinamide, 100 U/ml penicillin, and 100 mg/ml streptomycin and maintained at 37 °C (5% CO_2_, 95% air) in humidified air for 1, 2, 3 and 4 days. For NPCC xenotransplantation into nude mice, 3-day cultured NPCCs were centrifuged in PE-50 tubing connected to a 200 µL pipette tip. A capsulotomy was performed in the lower pole of the mouse kidney and the tip of the tubing was advanced under the capsule of the upper pole, the site of final injection. At 6, 9, 16, 23, 30 and 60 days post Tx, mice were anesthetized with amobarbital and an abdominal incision was made to expose kidneys. Under a dissecting microscope, the graft was removed, fixed and prepared as paraffin-embedded tissue blocks for histological analysis^9^.

### Semi-quantitative RT-PCR analysis

Total RNAs from NPCC culture were extracted using TRIzol reagent (Sigma) following the manufacturer’s instructions. Purified RNA concentration was measured by NanoDrop TM 1000 spectrophotometer. Reverse transcription was carried out using SuperScript III First-Strand Synthesis System (Invitrogen). Qualitative PCR reactions containing the mixture of cDNA, KAPA Taq ReadyMix, sense and antisense primers (Supplemental Table 1) were processed in a thermal cycler for indicated cycles. Samples were separated in 2% agarose gel and visualized using a Fuji imaging system.

### Quantitative histological analysis

Cultured NPCCs were pelleted, fixed with 4% PFA for 20–30 minutes and enrobed in agarose (w/v = 2%, Sigma) to make paraffin-embedded blocks for histological examination. The tissue blocks containing cultured NPCCs and NPCC-bearing grafts were sectioned by 5 micrometers and stained for indicated proteins. Antigen retrieval was performed in a PickCell 2100 antigen retriever in 10 mM citrate buffer solution (pH = 6.0). The primary and secondary fluorescent-conjugated (Alexafluor-488, −594, −647) antibodies (Supplemental Table 2) were sequentially applied onto the cells and DAPI was used as counter stain to define cell nuclei. Stained sections were examined using Leica TCS SP2 at Chang Gung Memorial Hospital. Final images were merged using Adobe Photoshop. The quantification analysis was performed from >5 randomly-selected areas per sample by 5 experienced investigators. All statistical analyses were performed using Microsoft Excel and statistical software Prism 5 (GraphPad) and presented as Mean ± SEM. Significant difference was defined as the p-value < 0.05.

### H2DCF-DA assay for ROS detection

For intracellular ROS analysis, isolated NPCCs were resuspended in PBS containing 2% FBS and loaded with 10 μM DCF-DA (Thermo Fisher Scientific) or 2.5 μM CellROX Deep Red (Thermo Fisher Scientific) and incubated at 37 °C for 30 minutes. An oxidation insensitive analog of Carboxy-DCFDA (Thermo Fisher Scientific) staining was used as a control to detect the probe uptake, probe efflux, and nonspecific probe activation. Cells were washed and resuspended in PBS containing 10 μM propidium iodide (PI) solution for analysis on a Beckman Coulter Cytomics FC500 Flow Cytometry at Instrumentation Resource Center (IRC), National Yang-Ming University. The protein concentration was used to normalize ROS level for 1- to 4-day NPCC culture.

## Electronic supplementary material

Supplemental Data
