# Supplementary material for: Porcine Neonatal Pancreatic Cell Clusters Maintain Their Multipotency in Culture and After Transplantation
Source: Sci Rep. 2018 May 29;8:8212. doi: 10.1038/s41598-018-26404-6 (PMC5974285; doi:10.1038/s41598-018-26404-6)
Supplement: Supplementary file 1 — Supplemental Data [file 41598_2018_26404_MOESM1_ESM.pdf]

Porcine Neonatal Pancreatic Cell Clusters Maintain Their Multipotency in Culture and After Transplantation

Wan-Chun Li, Chen-Yi Chen, Chen-Wei Kao, Pei-Chun Huang, Yi-Ta Hsieh, Tz-Yu Kuo, Tsai-Ying Chen, Hao-Yuan Chia, Jyuhn-Huarng Juang

Supplemental Materials

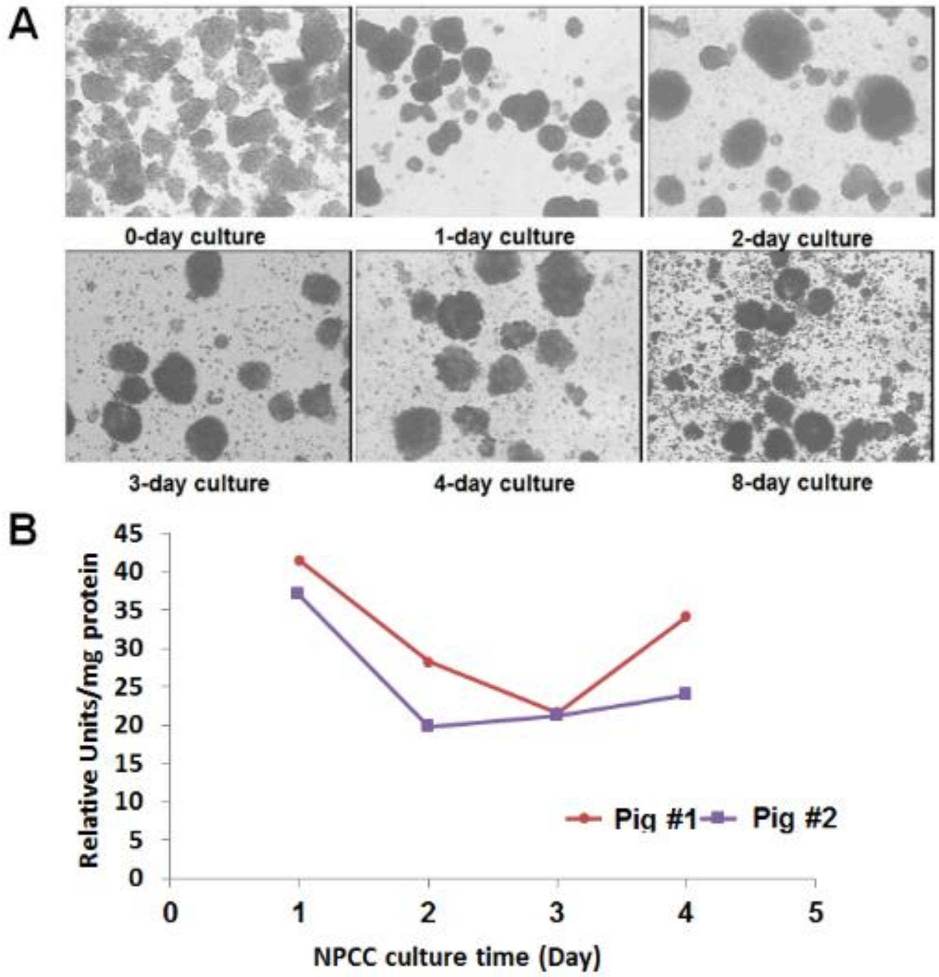

Supplemental Figure 1. (A) Microscopic analysis for NPCC culture. (B) The intracellular ROS level of NPCC cultures (N=2).

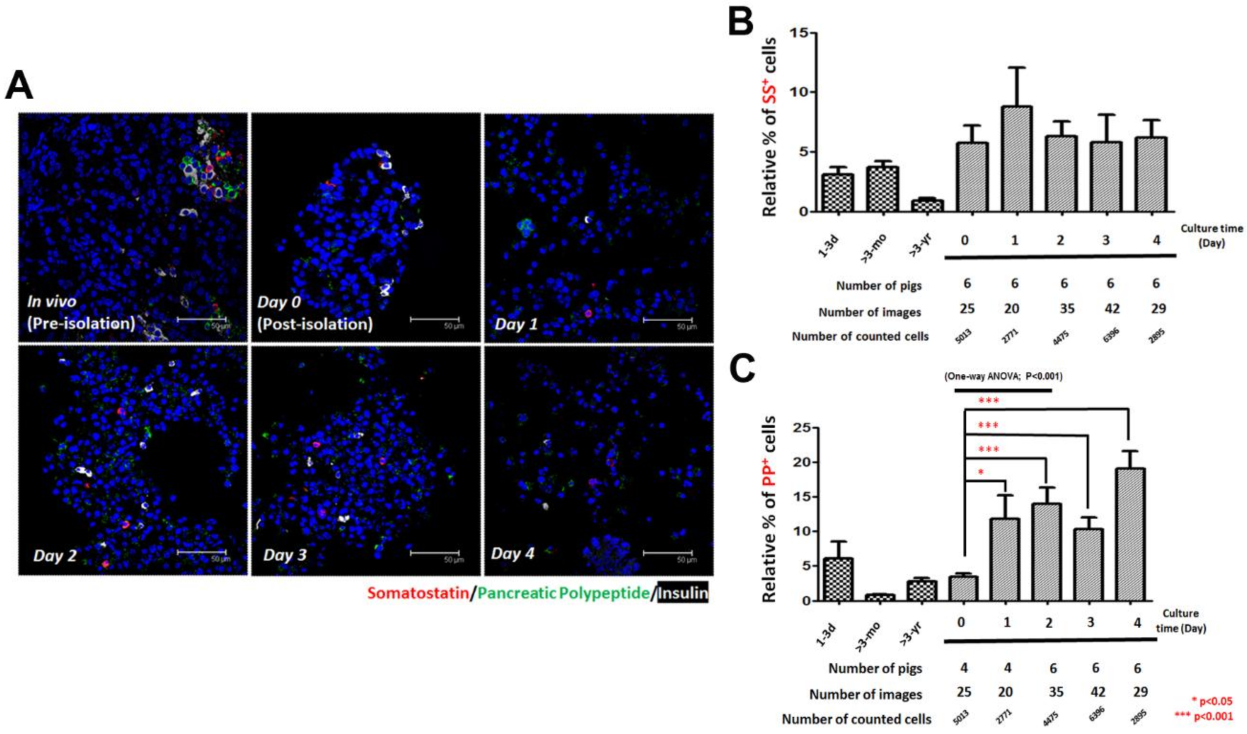

Supplemental Figure 2. Induction of somatostatin^{+} and pancreatic polypeptide^{+} cells in NPCC culture. IFA for (A) somatostatin (SS; green) / pancreatic polypeptide (PP; red) / insulin (white) in NPCC cultures. Quantitative results showed an increased (B) SS^{+} and (C) PP^{+} cell populations in NPCCs cultures over 4 days. DAPI is used to localize cell nuclei. *p<0.05, ***p<0.001.

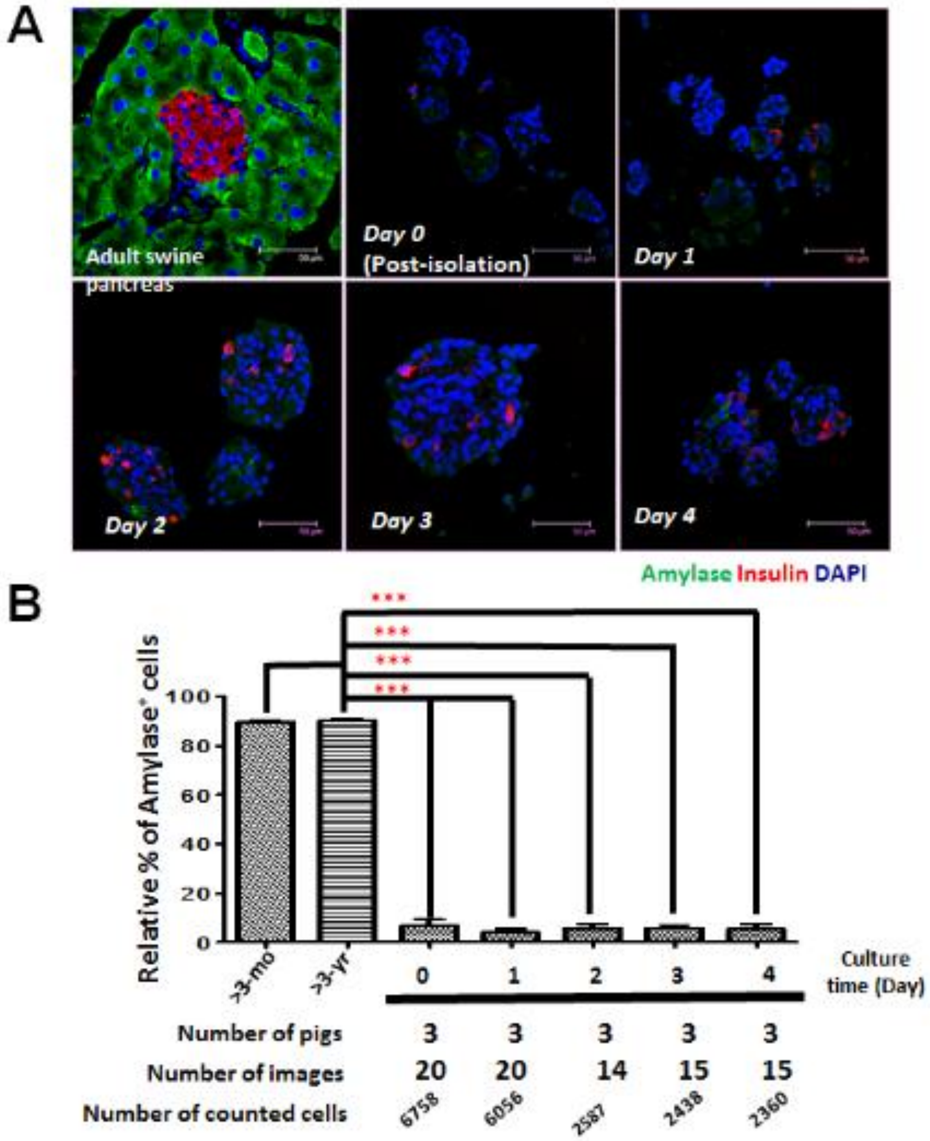

Supplemental Figure 3. Loss of amylase+ cells in NPCC culture. (A) IFA for amylase (green) and insulin (red) in NPCC cultures. Quantitative results showed a quick decrease of amylase+ cells in NPCCs cultures over 4 days. DAPI is used to localize cell nuclei. ***p<0.001.

.

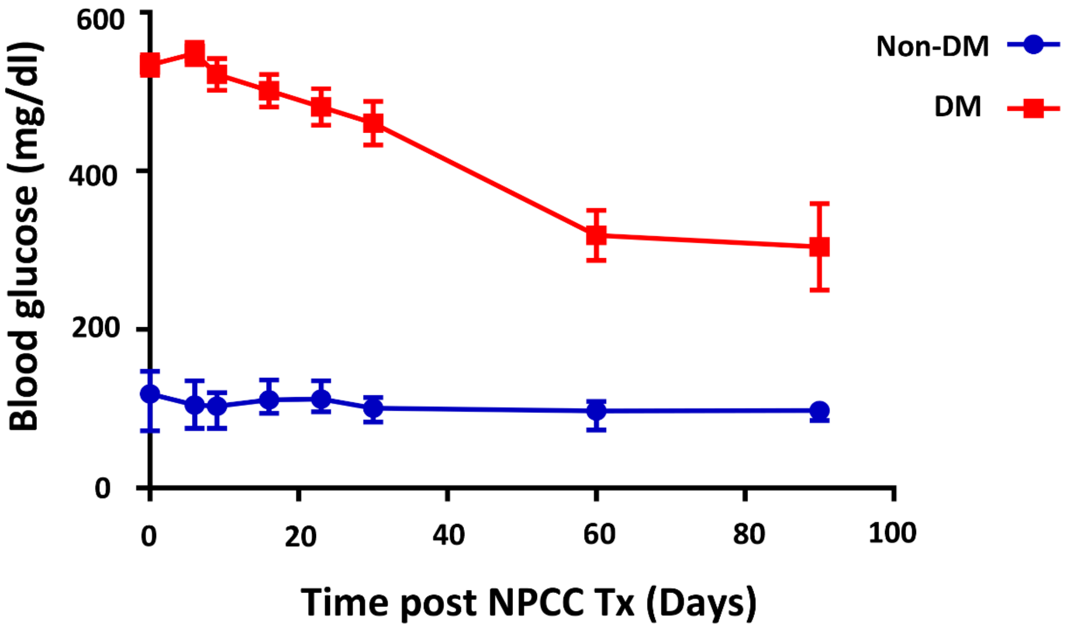

Supplemental Figure 4. Blood glucose levels of Non-DM and DM nude mice transplanted with 2,000 NPCCs.

Name	Forward primer	Reverse primer	Access #
Insulin	GCTTCTTCTACACGCCCAAG	CAGCACTGCTCCACGATG	NM_001109772
Glucagon	GAATTCATTGCTTGGCTGGT	CATCTGAGAAGGAGCCATCAG	NM_214324
Amylase(alpha 2B)	GCAAACTGGTCTTCCTGGTG	TGTGCCATCACTGGAAACAT	NM_214195
Pdx1	GTGGAAAAAGGAGGAGGACA	CAGCTCCTCTCCCGAGGT	NM_001141984
Sox9	CGCTCGCAGTACGACTACAC	CGGGTTCATGTAGGTGAAGG	NM_213843
CPB1	CCCGAGAACAATGCTGAGTT	TATCCCGGAGTTCAAAGGTG	NM_214169
RPL32	AGAAGGTTCAAGGGCCAGAT	TGCACATTAGCAGCACTTCA	NM_001001636

Supplemental Table 1. Primers sequences used in present study

Primary antibody	Cat. No	Vendor	Species
Insulin	Ab#ab7842	Abcam	Guinea pig
Glucagon	Dako#A0565	Dako	Rabbit
Ki67	Ab#ab92353	Abcam	Rabbit
SOX9	sc-20095	Santa Cruz	Rabbit
PDX1	Ab#ab47267	Abcam	Rabbit
E-cadherin	BD#610182	Abcam	Mouse
Pancreatic Polypeptide	SAB25500747	Sigma	Goat
Somatostatin	Ab#108456	Abcam	Rabbit

Secondary antibody	Vendor	Species
Anti-mouse HRP	Jackson Immuno Research	Donkey
Anti-rabbit HRP	Jackson Immuno Research	Donkey
Biotinylated anti-goat	Jackson Immuno Research	Rabbit
Alexa Fluor[R] 488 Anti-mouse	Jackson Immuno Research	Donkey
Alexa Fluor[R] 594 Anti-mouse	Jackson Immuno Research	Donkey
Alexa Fluor[R] 647 Anti-guinea pig	Abcam	Goat

Supplemental Table 2. Antibodies used in present study
